# Digital transcriptome profiling of normal and glioblastoma-derived neural stem cells identifies genes associated with patient survival

**DOI:** 10.1186/gm377

**Published:** 2012-10-09

**Authors:** Pär G Engström, Diva Tommei, Stefan H Stricker, Christine Ender, Steven M Pollard, Paul Bertone

**Affiliations:** 1 EMBL European Bioinformatics Institute, Wellcome Trust Genome Campus, Cambridge CB10 1SD, UK; 2 Samantha Dickson Brain Cancer Unit and Department of Cancer Biology, UCL Cancer Institute, University College London, Paul O'Gorman Building, 72 Huntley Street, London WC1E 6BT, UK; 3 Genome Biology and Developmental Biology Units, European Molecular Biology Laboratory, Meyerhofstraße 1, 69117 Heidelberg, Germany; 4 Wellcome Trust - Medical Research Council Cambridge Stem Cell Institute, University of Cambridge, Tennis Court Road, Cambridge CB2 1QR, UK

## Abstract

**Background:**

Glioblastoma multiforme, the most common type of primary brain tumor in adults, is driven by cells with neural stem (NS) cell characteristics. Using derivation methods developed for NS cells, it is possible to expand tumorigenic stem cells continuously *in vitro*. Although these glioblastoma-derived neural stem (GNS) cells are highly similar to normal NS cells, they harbor mutations typical of gliomas and initiate authentic tumors following orthotopic xenotransplantation. Here, we analyzed GNS and NS cell transcriptomes to identify gene expression alterations underlying the disease phenotype.

**Methods:**

Sensitive measurements of gene expression were obtained by high-throughput sequencing of transcript tags (Tag-seq) on adherent GNS cell lines from three glioblastoma cases and two normal NS cell lines. Validation by quantitative real-time PCR was performed on 82 differentially expressed genes across a panel of 16 GNS and 6 NS cell lines. The molecular basis and prognostic relevance of expression differences were investigated by genetic characterization of GNS cells and comparison with public data for 867 glioma biopsies.

**Results:**

Transcriptome analysis revealed major differences correlated with glioma histological grade, and identified misregulated genes of known significance in glioblastoma as well as novel candidates, including genes associated with other malignancies or glioma-related pathways. This analysis further detected several long non-coding RNAs with expression profiles similar to neighboring genes implicated in cancer. Quantitative PCR validation showed excellent agreement with Tag-seq data (median Pearson *r *= 0.91) and discerned a gene set robustly distinguishing GNS from NS cells across the 22 lines. These expression alterations include oncogene and tumor suppressor changes not detected by microarray profiling of tumor tissue samples, and facilitated the identification of a GNS expression signature strongly associated with patient survival (*P *= 1e-6, Cox model).

**Conclusions:**

These results support the utility of GNS cell cultures as a model system for studying the molecular processes driving glioblastoma and the use of NS cells as reference controls. The association between a GNS expression signature and survival is consistent with the hypothesis that a cancer stem cell component drives tumor growth. We anticipate that analysis of normal and malignant stem cells will be an important complement to large-scale profiling of primary tumors.

## Background

Glioblastoma (grade IV astrocytoma) is the most common and severe type of primary brain tumor in adults. The prognosis is poor, with a median survival time of 15 months despite aggressive treatment [1]. Glioblastomas display extensive cellular heterogeneity and contain a population of cells with properties characteristic of neural stem (NS) cells [2]. It has been proposed that such corrupted stem cell populations are responsible for maintaining cancers, and give rise to differentiated progeny that contribute to the cellular diversity apparent in many neoplasias. Data supporting this hypothesis have been obtained for several types of malignancies, including a variety of brain cancers [2]. Importantly, a recent study using a mouse model of glioblastoma demonstrated that tumor recurrence after chemotherapy originates from a malignant cell population with NS cell features [3]. Characterizing human glioblastoma cancer stem cells to understand how they differ from normal tissue stem cell counterparts may therefore provide key insights toward the identification of new therapeutic opportunities.

Fetal and adult NS cells can be isolated and maintained as untransformed adherent cell lines in serum-free medium supplemented with growth factors [4,5]. Using similar protocols, it is possible to expand NS cells from gliomas [6]. These glioma-derived NS (GNS) cells are very similar in morphology to normal NS cells, propagate continuously in culture and share expression of many stem and progenitor cell markers, such as SOX2 and Nestin. Like normal progenitor cells of the central nervous system, they can also differentiate into neurons, astrocytes and oligodendrocytes to varying degrees [5,6]. In contrast to NS cells, however, GNS cells harbor extensive genetic abnormalities characteristic of the disease and form tumors that recapitulate human gliomas when injected into mouse brain regions corresponding to sites of occurrence in patients.

In this study, we compare gene expression patterns of GNS and NS cells to discover transcriptional anomalies that may underlie tumorigenesis. To obtain sensitive and genome-wide measurements of RNA levels, we conducted high-throughput sequencing of transcript tags (Tag-seq) on GNS cell lines from three glioblastoma cases and on two normal NS cell lines, followed by quantitative reverse transcription PCR (qRT-PCR) validation in a large panel of GNS and NS cell lines. Tag-seq is an adaptation of serial analysis of gene expression (SAGE) to high-throughput sequencing and has considerable sensitivity and reproducibility advantages over microarrays [7,8]. Compared to transcriptome shotgun sequencing (RNA-seq), Tag-seq does not reveal full transcript sequences, but has the advantages of being strand-specific and unbiased with respect to transcript length.

A large body of microarray expression data for glioblastoma biopsies has been generated through multiple studies [9-13]. These data have been extensively analyzed to detect gene expression differences among samples, with the aim to identify outliers indicative of aberrant expression [11,14,15], discover associations between gene expression and prognosis [12,16] or classify samples into clinically relevant molecular subtypes [9,10,13,17]. However, expression profiling of tumor specimens is limited by the inherent cellular heterogeneity of malignant tissue and a lack of reference samples with similar compositions of corresponding normal cell types. GNS cells represent a tractable alternative for such analyses, as they constitute a homogeneous and self-renewing cell population that can be studied in a wide range of experimental contexts and contrasted with genetically normal NS cells. By combining the sensitive Tag-seq method with the GNS/NS model system we obtain a highly robust partitioning of malignant and normal cell populations, and identify candidate oncogenes and tumor suppressors not previously associated with glioma.

## Materials and methods

### Cell culture and sample preparation

GNS and NS cells were cultured in N2B27 serum-free medium [18], a 1:1 mixture of DMEM/F-12 and Neurobasal media (Invitrogen, Paisley, UK) augmented with N2 (Stem Cell Sciences, Cambridge, UK) and B27 (Gibco, Paisley, UK) supplements. Self-renewal was supported by the addition of 10 ng/ml epidermal growth factor and 20 ng/ml fibroblast growth factor 2 to the complete medium. Cells were plated at 20,000/cm^2 ^in laminin-coated vessels (10 μg/ml laminin-1 (Sigma, Dorset, UK) in phosphate-buffered saline for 6 to 12 h), passaged near confluence using Accutase dissociation reagent (Sigma) and were typically split at 1:3 for NS cells and 1:3 to 1:6 for GNS cells. For expression analysis, cells were dissociated with Accutase and RNA was extracted using RNeasy (Qiagen, West Sussex, UK), including a DNase digestion step. RNA quality was assessed on the 2100 Bioanalyzer (Agilent, Berkshire, UK).

### Transcriptome tag sequencing

Tag-seq entails the capture of polyadenylated RNA followed by extraction of a 17-nucleotide (nt) sequence immediately downstream of the 3′-most NlaIII site in each transcript. These 17 nt 'tags' are sequenced in a high-throughput manner and the number of occurrences of each unique tag is counted, resulting in digital gene expression profiles where tag counts reflect expression levels of corresponding transcripts [8].

Tag-seq libraries were prepared using the Illumina NlaIII DGE protocol. Briefly, polyadenylated RNA was isolated from 2 µg total RNA using Sera-Mag oligo(dT) beads (Thermo Scientific, Leicestershire, UK). First-strand cDNA was synthesized with SuperScript II reverse transcriptase (Invitrogen) for 1 h at 42°C, followed by second-strand synthesis by DNA polymerase I for 2.5 h at 16°C in the presence of RNase H. cDNA products were digested with NlaIII for 1 h at 37°C and purified to retain only the 3′-most fragments bound to the oligo(dT) beads. Double-stranded GEX adapter 1 oligonucleotides, containing an MmeI restriction site, were ligated to NlaIII digestion products with T4 DNA ligase for 2 h at 20°C. Ligation products were then digested with MmeI at the adapter-cDNA junction site, thereby creating 17 bp tags free in solution. GEX adapter 2 oligos were ligated to the MmeI cleavage site by T4 DNA ligase for 2 h at 20°C, and the resulting library constructs were PCR-amplified for 15 cycles with Phusion DNA polymerase (Finnzymes, Essex, UK).

Libraries were sequenced at Canada's Michael Smith Genome Sciences Centre, Vancouver BC on the Illumina platform. Transcript tags were extracted as the first 17 nt of each sequencing read and raw counts obtained by summing the number of reads for each observed tag. To correct for potential sequencing errors, we used the *Recount *program [19], setting the Hamming distance parameter to 1. *Recount *uses an expectation maximization algorithm to estimate true tag counts (that is, counts in the absence of error) based on observed tag counts and base-calling quality scores. Tags matching adapters or primers used in library construction and sequencing were identified and excluded using *TagDust *[20] with a target false discovery rate (FDR) of 1%. Tags derived from mitochondrial or ribosomal RNA were identified and excluded by running the *bowtie *short-read aligner [21] against a database consisting of all ribosomal RNA genes from Ensembl [22], all ribosomal repeats in the UCSC Genome Browser RepeatMasker track for genome assembly GRCh37 [23], and the mitochondrial DNA sequence; only perfect matches to the extended 21 nt tag sequence (consisting of the NlaIII site CATG followed by the observed 17 nt tag) were accepted. Remaining tags were assigned to genes using a hierarchical strategy based on the expectation that tags are most likely to originate from the 3'-most NlaIII site in known transcripts (Additional files 1 and 2). To this end, expected tag sequences (virtual tags) were extracted from the SAGE Genie database [24] and Ensembl transcript sequences. In addition, *bowtie *was applied to determine unique, perfect matches for sequenced tags to the reference genome.

The Bioconductor package *DESeq *[25] was used to normalize tag counts, call differentially expressed genes and obtain variance-stabilized expression values for correlation calculations. Tests for enrichment of Gene Ontology and InterPro terms were performed in R, using Gene Ontology annotation from the core Bioconductor package *org.Hs.eg *and InterPro annotation from Ensembl. Each term associated with a gene detected by Tag-seq was tested. Signaling pathway impact analysis was carried out using the Bioconductor package *SPIA *[26]. To identify major differences common to the GNS cell lines investigated, we filtered the set of genes called differentially expressed at 1% FDR, further requiring (i) two-fold or greater change in each GNS cell line compared to each NS cell line, with the direction of change being consistent among them; and (ii) expression above 30 tags per million in each GNS cell line (if upregulated in GNS cells) or each NS cell line (if downregulated in GNS cells). Sequencing data and derived gene expression profiles are available from ArrayExpress [27] under accession E-MTAB-971.

### Quantitative RT-PCR validation

Custom-designed TaqMan low-density array microfluidic cards (Applied Biosystems, Paisley, UK) were used to measure the expression of 93 genes in 22 cell lines by qRT-PCR. This gene set comprises 82 validation targets from Tag-seq analysis, eight glioma and developmental markers, and three endogenous control genes (18S ribosomal RNA, *TUBB *and *NDUFB10*). The 93 genes were interrogated using 96 different TaqMan assays (three of the validation targets required two different primer and probe sets to cover all known transcript isoforms matching differentially expressed tags). A full assay list with raw and normalized threshold cycle (C*_t_*) values is provided in Additional file 3. To capture biological variability within cell lines, we measured up to four independent RNA samples per line. cDNA was generated using SuperScript III (Invitrogen) and real-time PCR carried out using TaqMan fast universal PCR master mix. C*_t _*values were normalized to the average of the three control genes using the Bioconductor package *HTqPCR *[28]. Differentially expressed genes were identified by the Wilcoxon rank sum test after averaging replicates.

### Tumor gene expression analysis

Public microarray data, survival information and other associated metadata were obtained from The Cancer Genome Atlas (TCGA) and four independent studies (Table 1). All tumor microarray data were from samples obtained upon initial histologic diagnosis. We used processed (level 3) data from TCGA, consisting of one expression value per gene and sample (Additional file 4). For the other data sets, we processed the raw microarray data with the RMA method in the Bioconductor package *affy *[29] and retrieved probe-gene mappings from Ensembl 68 [22]. For genes represented by multiple probesets, expression values were averaged across probesets for randomization tests, heatmap visualization and GNS signature score calculation. Differential expression was computed using *limma *[30]. Randomization tests were conducted with the *limma *function geneSetTest, comparing log_2 _fold-change for core up- or downregulated genes against the distribution of log_2 _fold-change for randomly sampled gene sets of the same size.

**Table 1:** Public gene expression data sets used in this study

			Number of cases				
Citation	Citation	Microarray platform (Affymetrix)	Glioblastoma	Grade III astrocytoma	Other grade III glioma	Grade I-II glioma	Non-neoplastic brain
The Cancer Genome Atlas (TCGA) [11,46]	NA	Exon 1.0 ST	397	0	0	0	10
Gravendeel *et al. *[13]	GSE16011	U133 Plus 2.0	141	16	66	27	0
Murat *et al. *[12]	GSE7696	U133 Plus 2.0	70	0	0	0	0
Phillips *et al. *[9]	GSE4271	U133A and U133B	55	21	0	0	0
Freije *et al. *[10]	GSE4412	U133A and U133B	50	8	16	0	0

TCGA sample IDs are listed in Additional file 4. Gravendeel *et al. *[13] described 269 samples obtained at histologic diagnosis, from which we excluded 15 containing mostly non-neoplastic tissue and four lacking survival data. NA, not applicable.

Survival analysis was carried out with the R library *survival*. To combine expression values of multiple genes for survival prediction, we took an approach inspired by Colman *et al. *[16]. The normalized expression values *x_ij_*, where *i *represents the gene and *j *the sample, were first standardized to be comparable between genes by subtracting the mean across samples and dividing by the standard deviation, thus creating a matrix of *z*-scores:

$$z_{ij}=\frac{x_{ij}-{\bar{x}}_{i.}}{\text{SD}\left(x_{i.}\right)}$$

Using a set *U *of *n_U _*genes upregulated in GNS cell lines and a set *D *of *n_D _*genes downregulated in these cells, we then computed a GNS signature score *s_j _*for each sample *j *by subtracting the mean expression of the downregulated genes from the mean expression of the upregulated genes:

$$s_{j}=\underset{i\in U}{\sum }\frac{z_{ij}}{n_{U}}-\underset{i\in D}{\sum }\frac{z_{ij}}{n_{D}}$$

*IDH1 *mutation calls for TCGA samples were obtained from Firehose data run version 2012-07-07 [31] and data files from the study by Verhaak *et al. *updated 2011-11-28 [32].

### Array comparative genomic hybridization

We re-analyzed the array comparative genomic hybridization (CGH) data described by Pollard *et al. *[6]. CGH was performed with Human Genome CGH Microarray 4x44K arrays (Agilent), using genomic DNA from each cell line hybridized in duplicate (dye swap) and normal human female DNA as reference (Promega, Southampton, UK). Log_2 _ratios were computed from processed Cy3 and Cy5 intensities reported by the software *CGH Analytics *(Agilent). We corrected for effects related to GC content and restriction fragment size using a modified version of the waves array CGH correction algorithm [33]. Briefly, log_2 _ratios were adjusted by sequential loess normalization on three factors: fragment GC content, fragment size, and probe GC content. These were selected after investigating dependence of log ratio on multiple factors, including GC content in windows of up to 500 kb centered around each probe. The Bioconductor package *CGHnormaliter *[34] was then used to correct for intensity dependence and log_2 _ratios scaled to be comparable between arrays using the 'scale' method in the package *limma *[35]. Replicate arrays were averaged and the genome (GRCh37) segmented into regions with different copy number using the circular binary segmentation algorithm in the Bioconductor package *DNAcopy *[36], with the option undo.SD set to 1. Aberrations were called using the package *CGHcall *[37] with the option nclass set to 4. CGH data are available from ArrayExpress [27] under accession E-MTAB-972.

## Results

### Transcriptome analysis highlights pathways affected in glioma

We applied Tag-seq to four GNS cell lines (G144, G144ED, G166 and G179) and two human fetal NS cell lines (CB541 and CB660), all previously described [5,6]. G144 and G144ED were independently established from the same parental tumor in different laboratories. Tag-seq gene expression values were strongly correlated between these two lines (Pearson *r *= 0.94), demonstrating that the experimental procedure, including cell line establishment, library construction and sequencing, is highly reproducible. The two NS cell transcriptome profiles were also well correlated (*r *= 0.87), but there were greater differences among G144, G166 and G179 (*r *ranging from 0.78 to 0.82). This is expected, as G144, G166 and G179 originate from different and histologically distinct glioblastoma cases.

**Figure 1:**
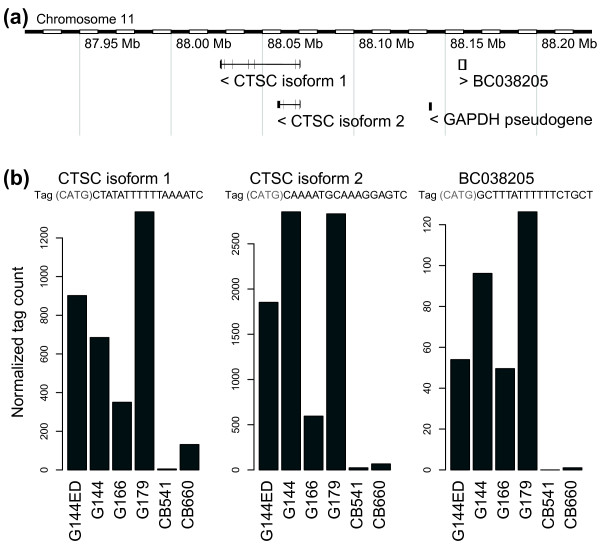
**Correlated expression of *CTSC *and a nearby non-coding RNA**. **(a) ***CTSC *(cathepsin C) is located in a gene desert harboring an uncharacterized non-coding gene transcribed in the opposite orientation [GenBank:BC038205]. **(b) **Both *CTSC *and the non-coding RNA have strongly elevated expression in GNS relative to NS cell lines, with highest levels in G179.

We used the Tag-seq data to identify differences in gene expression between the three GNS cell lines G144, G166 and G179 and the two normal NS cell lines CB541 and CB660. At a FDR of 10%, this analysis revealed 485 genes to be expressed at a higher average level in GNS cells (upregulated) and 254 genes to be downregulated (Additional file 5). GNS cells display transcriptional alterations common in glioblastoma, including upregulation of the epidermal growth factor receptor (*EGFR*) gene and downregulation of the tumor suppressor *PTEN *[11]. Enrichment analysis using Gene Ontology and the KEGG (Kyoto Encyclopedia of Genes and Genomes) pathway database confirmed the set of 739 differentially expressed genes to be enriched for pathways related to brain development, glioma and cancer (Tables 2 and 3). We also observed enrichment of regulatory and inflammatory genes, such as signal transduction components, cytokines, growth factors and DNA-binding factors. Several genes related to antigen presentation on MHC class I and II molecules were upregulated in GNS cells, consistent with the documented expression of their corresponding proteins in glioma tumors and cell lines [38,39]. In addition, we detected 25 differentially expressed long non-coding RNAs (Additional file 6). Several of these display an expression pattern similar to a neighboring protein-coding gene, including cancer-associated genes *DKK1 *and *CTSC *[40,41] (Figure 1) and developmental regulators *IRX2*, *SIX3 *and *ZNF536 *[42], suggesting that they may be functional RNAs regulating nearby genes [43] or represent transcription from active enhancers [44].

**Table 2:** Selected Gene Ontology terms and InterPro domains enriched among differentially expressed genes

	Differentially expressed (729 genes)		Upregulated (485 genes)		Downregulated (254 genes)	
	Genes	*P*	Genes	*P*	Genes	*P*
**Biological process Gene Ontology terms**						
Immune response	70	2.4 × 10^-12^	61	3.0 × 10^-16^	9	NS
Nervous system development	106	1.9 × 10^-10^	62	0.0055	44	2.3 × 10^-5^
Cell adhesion	74	9.8 × 10^-8^	56	1.9 × 10^-7^	18	NS
Antigen processing and presentation	17	4.3 × 10^-7^	17	5.4 × 10^-10^	0	NS
Cell differentiation	128	7.4 × 10^-7^	74	NS	54	1.8 × 10^-4^
Cell migration	44	3.0 × 10^-4^	30	0.0262	14	NS
Cell proliferation	86	3.4 × 10^-4^	59	0.0136	27	NS
Cellular ion homeostasis	36	0.0138	33	1.7 × 10^-5^	3	NS
						
**Molecular function Gene Ontology terms**						
Cytokine activity	27	2.3 × 10^-8^	25	7.1 × 10^-11^	2	NS
Signal transducer activity	111	2.8 × 10^-7^	66	0.0584	45	0.0017
Receptor activity	83	8.0 × 10^-7^	48	NS	35	0.0016
Sequence-specific DNA binding	52	2.7 × 10^-4^	34	0.0526	18	NS
MHC class II receptor activity	5	0.0077	5	9.2 × 10^-4^	0	NS
Growth factor activity	20	0.0109	17	0.0019	3	NS
						
**InterPro domains**						
Immunoglobulin-like	45	3.1 × 10^-8^	32	6.0 × 10^-6^	13	NS
MHC classes I/II-like antigen recognition protein	14	1.1 × 10^-7^	14	3.2 × 10^-10^	0	NS
Homeobox	28	8.5 × 10^-6^	18	0.0124	10	NS

Number of genes differentially expressed at 10% FDR and annotated with the indicated Gene Ontology and InterPro terms. *P*-values indicating the statistical significance of enrichment of these terms were computed with Fisher's exact test and corrected for multiple testing using the Bonferroni method. NS, not significant (*P *> 0.1).

**Table 3:** Representative KEGG pathways from signaling pathway impact analysis of gene expression differences between GNS and NS cell lines

Pathway	Genes	*P*	Predicted status in GNS cells
Cytokine-cytokine receptor interaction	29	4.4 × 10^-12^	Activated
Chemokine signaling pathway	15	5.3 × 10^-6^	Activated
Neuroactive ligand-receptor interaction	21	2.2 × 10^-4^	Inhibited
Antigen processing and presentation	11	6.8 × 10^-4^	Activated
MAPK signaling pathway	24	0.0106	Activated
Glioma	10	0.0131	Activated
ECM-receptor interaction	10	0.0405	Inhibited
Calcium signaling pathway	15	0.0405	Activated

The number of genes found to be differentially expressed at 10% FDR and belonging to the selected pathways are indicated. *P*-values and status predictions were obtained by signaling pathway impact analysis [26], taking fold-change estimates and pathway topology into account. *P*-values were FDR-corrected for multiple testing. ECM, extracellular matrix; MAPK, mitogen-activated protein kinase.

**Figure 2:**
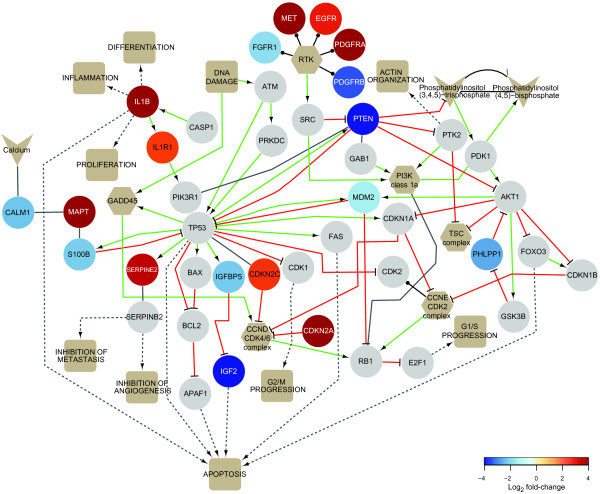
**Expression changes in pathways most commonly affected in glioma**. Genes are represented by circles and colored according to fold-change between GNS and NS cells measured by Tag-seq (see color key), or grey unless statistically significant (10% FDR). Gene complexes and families (hexagons), small molecules (chevrons) and affected cellular processes (squares) are included. Edges indicate activation (green), inhibition (red), contains (black with circular tip), becomes (black with half arrow) and other interactions (grey).

To visualize gene expression differences in a pathway context, we compiled an integrated pathway map that includes the pathways most commonly affected in glioblastoma, as well as pathways related to antigen processing and presentation, apoptosis, angiogenesis and invasion (Additional file 1). The map contains 182 genes, of which 66 were differentially expressed between GNS and NS cells at 10% FDR (Additional file 7). Figure 2 depicts a condensed version focused on the pathways most frequently affected in glioblastoma. This approach allowed us to identify differentially expressed genes that participate in glioma-related pathways, but have not been directly implicated in glioma. These include several genes associated with other neoplasms (Table 4). Our comparison between GNS and NS cells thus highlights genes and pathways that are known to be affected in glioma as well as novel candidates, and suggests the GNS/NS comparison is a compelling model for investigating the molecular attributes of glioma.

**Table 4:** Novel candidate glioma genes identified by differential expression and pathway analysis

Gene^a^	Log_2 _fold-change^b^	Prior association with glioma	Implication in other neoplasms	Reference
*CACNA1A*	7.1	None	Prostate cancer (mouse model)	[80]
*CACNA1C*	-8.2	None	Liver cancer	[81]
*CACNG7*	-2.6	None	None	--
*CACNG8*	-4.8	None	None	--
*CAMK1D*	-2.4	None	Breast cancer	[82]
*CPLX2*	6.4	None	None	--
*DDIT3 *(*CHOP*, *GADD153*)	4.4	Limited	General (cellular stress response)	[83-86]
*DUSP16*	4.2	None	Burkitt's lymphoma	[87]
*FGF19*	-	None	Liver, lung and colon cancer	[88]
*ITGA4 *(*CD49D*)	3.0	Limited	Chronic lymphocytic leukemia, breast cancer and others	[89-91]
*ITGBL1*	+	None	None	--
*MAP3K5 *(*ASK1*)	5.1	Limited	Gastric cancer and histiocytoma	[92-94]
*NFATC2 *(*NFAT1*)	+	Limited	Breast cancer	[95-98]
*NFKBIZ*	5.1	None	Liposarcoma	[99]
*NR0B1 *(*DAX1*)	+	None	Lung adenocarcinoma and Ewing's sarcoma	[100,101]
*NR1D1*	2.9	None	Breast cancer	[102]
*PARP3*, *PARP12*	4.1, 2.9	By homology^c^	The PARP gene family is involved in DNA repair and several other processes related to tumorigenesis	[103,104]
*PERP*	3.8	None	Lung and skin cancer	[105,106]
*PPEF1*	4.4	Limited	None	[107]
*SNAP25*	3.3	None	Lung cancer	[108]
*SYT1*	-2.5	None	None	--
*TNFRSF14*	4.0	None	Follicular lymphoma	[109]
*TNFSF4 *(*OX40L*)	4.0	None	Generally implicated in immune response to tumors	[110]

^a^Aliases are listed in parentheses. ^b^Gene expression log_2 _fold-change between GNS and NS cell lines compared by Tag-seq. Some genes were detected exclusively in GNS or NS cells (indicated in column 2 by + or -, respectively). ^c^The homolog *PARP1 *has been implicated in glioma. PARP, poly(ADP-ribose) polymerase.

### Core expression changes in GNS lines are mirrored in glioma tumors and correlate with histological grade

**Figure 3:**
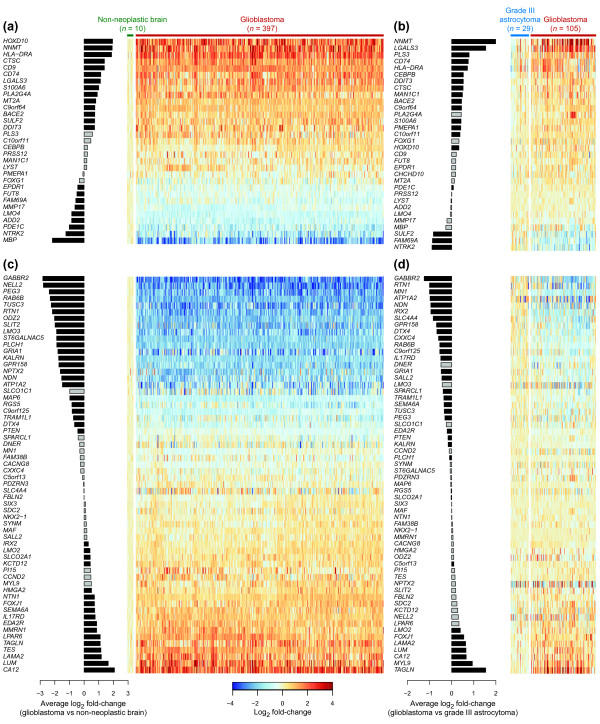
**Core gene expression changes in GNS lines are mirrored in glioblastoma tumors**. **(a-d) **Expression in tumors for genes that are strongly upregulated (a,b) or downregulated (c,d) in GNS cells. The gene sets were identified by comparison of Tag-seq expression profiles for GNS and NS cell lines (see main text). Bars depict average fold-change between glioblastoma and non-neoplastic brain tissue (a,c) (TCGA data set) and between glioblastoma and grade III astrocytoma (b,d) (Phillips and Freije data sets combined). Black bars indicate genes with significant differential expression in the microarray data (*P *< 0.01). Heatmaps show expression in individual samples relative to the average in non-neoplastic brain (a,c) or grade III astrocytoma (b,d). One gene (*CHCHD10*) not quantified in the TCGA data set is omitted from (a).

To capture major gene expression changes common to G144, G166 and G179, we set strict criteria on fold changes and tag counts (see Materials and methods). This approach yielded 32 upregulated and 60 downregulated genes, in the following referred to as 'core' differentially expressed genes (Additional file 8). This set includes genes with established roles in glioblastoma (for example, *PTEN *[11] and *CEBPB *[45]), as well as others not previously implicated in the disease (see Discussion). To investigate whether these core differentially expressed genes have similar expression patterns in GNS cells and primary tumors, we made use of public microarray data (Table 1). Perfect agreement between tissue- and cell-based results would not be expected, as tissues comprise a heterogeneous mixture of cell types. Nevertheless, analysis of microarray expression data from TCGA [11,46] for 397 glioblastoma cases (Additional file 4) revealed a clear trend for core upregulated GNS genes to be more highly expressed in glioblastoma tumors than in non-neoplastic brain tissue (*P *= 0.02, randomization test; Figure 3a) and an opposite trend for core downregulated genes (*P *= 3 × 10^-5^; Figure 3c).

We hypothesized that the expression of these genes might also differ between glioblastoma and less severe astrocytomas. We therefore examined their expression patterns in microarray data from the studies of Phillips *et al. *[9] and Freije *et al. *[10], which both profiled grade III astrocytoma cases in addition to glioblastomas (Table 1). The result was similar to the comparison with non-neoplastic brain tissue above; there was a propensity for core upregulated genes to be more highly expressed in glioblastoma than in the lower-grade tumor class (*P *= 10^-6^; Figure 3b), while core downregulated genes showed the opposite pattern (*P *= 10^-4^; Figure 3d). The set of core differentially expressed genes identified by Tag-seq thus defines an expression signature characteristic of glioblastoma and related to astrocytoma histological grade.

### Large-scale qRT-PCR validates Tag-seq results and identifies a robust gene set distinguishing GNS from NS cells

**Figure 4:**
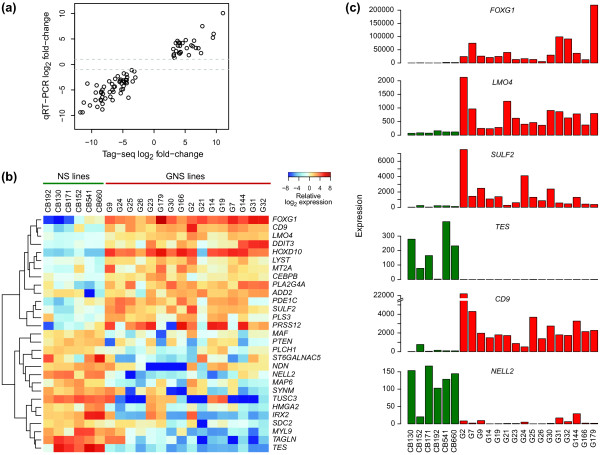
**qRT-PCR validates Tag-seq results and identifies a robust gene set distinguishing GNS from NS cells**. **(a) **Fold-change estimates (indicating expression level in GNS relative to NS cell lines) from Tag-seq and qRT-PCR for each of the 82 genes measured by qRT-PCR. Greater than two-fold difference in expression (dashed lines at *y *= ±1) was confirmed for all genes. **(b) **Heatmap of 29 genes differentially expressed between 16 GNS and six NS cell lines. Colors indicate qRT-PCR ΔΔC*_t _*values, that is, normalized expression on a log_2 _scale, where zero corresponds to the average expression between the two groups (GNS and NS cells). **(c) **Expression levels of the top six genes (by Wilcoxon test *P*-value) distinguishing GNS from NS cell lines, measured by qRT-PCR and presented as percentage of NS geometric mean.

To assess the accuracy of Tag-seq expression level estimates and investigate gene activity in a larger panel of cell lines, we assayed 82 core differentially expressed genes in 16 GNS cell lines (derived from independent patient tumors) and six normal NS cell lines by qRT-PCR using custom-designed TaqMan microfluidic arrays. The 82 validation targets (Additional file 3) were selected from the 92 core differentially expressed genes based on the availability of TaqMan probes and considering prior knowledge of gene functions. For the cell lines assayed by both Tag-seq and qRT-PCR, measurements agreed remarkably well between the two technologies: the median Pearson correlation for expression profiles of individual genes was 0.91 and the differential expression calls were corroborated for all 82 genes (Figure 4a). Across the entire panel of cell lines, 29 of the 82 genes showed statistically significant differences between GNS and NS cells at an FDR of 5% (Figure 4b,c). This set of 29 genes generally distinguishes GNS cells from normal NS cell counterparts, and may therefore have broad relevance for elucidating properties specific to tumor-initiating cells.

### A GNS cell expression signature is associated with patient survival

To further explore the relevance in glioma for these recurrent differences between GNS and NS cell transcriptomes, we integrated clinical information with tumor expression data. We first tested for associations between gene expression and survival time using the TCGA data set consisting of 397 glioblastoma cases (Table 1). For each gene, we fitted a Cox proportional hazards model with gene expression as a continuous explanatory variable and computed a *P*-value by the score test (Table 5). The set of 29 genes found to distinguish GNS from NS cells across the 22 cell lines assayed by qRT-PCR was enriched for low *P*-values compared to the complete set of 18,632 genes quantified in the TCGA data set (*P *= 0.02, one-sided Kolmogorov-Smirnov test), demonstrating that expression analysis of GNS and NS cell lines had enriched for genes associated with patient survival. Seven of the 29 genes had a *P*-value below 0.05 and, for six of these, the direction of the survival trend was concordant with the expression in GNS cells, such that greater similarity to the GNS cell expression pattern indicated poor survival. Specifically, *DDIT3*, *HOXD10*, *PDE1C *and *PLS3 *were upregulated in GNS cells and expressed at higher levels in glioblastomas with poor prognosis, while *PTEN *and *TUSC3 *were downregulated in GNS cells and expressed at lower levels in gliomas with poor prognosis.

**Table 5:** Survival tests for 29 genes distinguishing GNS from NS lines

		TCGA data set		Gravendeel data set (glioblastoma cases)		
Gene	Category	Coefficient^a^	*P*	Probeset^b^	Coefficient^a^	*P*
*ADD2*	Upregulated	-0.13	0.2858	237336_at	-0.17	0.1420
*CD9*	Upregulated	0.18	0.0731	201005_at	0.17	0.0689
*CEBPB*	Upregulated	0.19	0.1028	212501_at	0.17	0.0651
*DDIT3*	Upregulated	0.17	0.0128	209383_at	0.09	0.2777
*FOXG1*	Upregulated	0.13	0.0861	206018_at	0.11	0.0380
*HMGA2*	Downregulated	0.13	0.1456	1561633_at	-0.84	0.2459
*HOXD10*	Upregulated	0.12	0.0108	229400_at	0.15	0.0021
*IRX2*	Downregulated	-0.19	0.2346	228462_at	-0.20	4.4 × 10^-4^
*LMO4*	Upregulated	0.24	0.1046	209205_s_at	0.20	0.1435
*LYST*	Upregulated	0.05	0.5590	203518_at	0.10	0.4151
*MAF*	Downregulated	0.10	0.5873	209348_s_at	0.38	0.0074
*MAP6*	Downregulated	0.16	0.3063	235672_at	-0.30	0.0087
*MT2A*	Upregulated	0.16	0.1554	212185_x_at	0.27	0.0127
*MYL9*	Downregulated	0.08	0.3764	201058_s_at	0.15	0.0252
*NDN*	Downregulated	-0.04	0.4874	209550_at	-0.22	6.0 × 10^-5^
*NELL2*	Downregulated	0.08	0.1021	203413_at	0.14	0.0215
*PDE1C*	Upregulated	0.20	0.0105	236344_at	0.21	0.0134
*PLA2G4A*	Upregulated	-0.06	0.3198	210145_at	0.30	2.9 × 10^-4^
*PLCH1*	Downregulated	0.10	0.3165	214745_at	0.45	0.0094
*PLS3*	Upregulated	0.13	0.0381	201215_at	0.30	0.0069
*PRSS12*	Upregulated	-0.11	0.1865	213802_at	0.20	0.0296
*PTEN*	Downregulated	-0.53	0.0047	228006_at	-0.40	0.0062
*SDC2*	Downregulated	0.22	0.0044	212158_at	0.28	5.8 × 10^-4^
*ST6GALNAC5*	Downregulated	0.01	0.9116	220979_s_at	0.08	0.2416
*SULF2*	Upregulated	-0.11	0.1525	233555_s_at	-0.15	0.0930
*SYNM*	Downregulated	-0.06	0.5620	212730_at	0.08	0.2613
*TAGLN*	Downregulated	0.03	0.5947	205547_s_at	0.17	0.0030
*TES*	Downregulated	-0.05	0.5759	202720_at	0.07	0.5499
*TUSC3*	Downregulated	-0.14	0.0079	209227_at	-0.18	0.0060

^a^Fitted coefficient from Cox model; a positive coefficient indicates that higher expression is associated with poor survival and a negative coefficient indicates the opposite. ^b^For the Gravendeel data set, the result for the most significant probeset interrogating the gene is shown.

We reasoned that, if a cancer stem cell subpopulation in glioblastoma tumors underlies these survival trends, it may be possible to obtain a stronger and more robust association with survival by integrating expression information for multiple genes up- or downregulated in GNS cells. We therefore combined the expression values for the genes identified above (*DDIT3*, *HOXD10*, *PDE1C*, *PLS3*, *PTEN *and *TUSC3*) into a single value per tumor sample, termed 'GNS signature score' (see Materials and methods). This score was more strongly associated with survival (*P *= 10^-6^) than were the expression levels of any of the six individual genes (*P *ranging from 0.005 to 0.04; Table 5).

**Figure 5:**
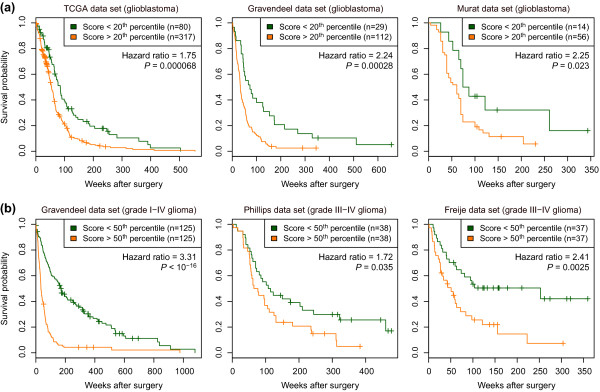
**Association between GNS signature score and patient survival**. **(a,b) **Kaplan-Meier plots illustrate the association between signature score and survival for three independent glioblastoma data sets (a) and three data sets that include gliomas of lower grade (b) (Table 1). Higher scores indicate greater similarity to the GNS cell expression profile. Hazard ratios and log-rank *P*-values were computed by fitting a Cox proportional hazards model to the data. Percentile thresholds were chosen for illustration; the association with survival is statistically significant across a wide range of thresholds (Additional file 9) and the *P*-values given in the text and Table 6 were computed without thresholding, using the score as a continuous variable.

To test whether these findings generalize to independent clinical sample groups, we examined the glioblastoma data sets described by Gravendeel *et al. *[13] and Murat *et al. *[12], consisting of 141 and 70 cases, respectively (Table 1). The GNS signature score was correlated with patient survival in both of these data sets (*P *= 3 × 10^-5 ^and 0.006, respectively; Figure 5a; Additional file 9). At the level of individual GNS signature genes, five were significantly associated with survival (*P *< 0.05) in both of the two largest glioblastoma data sets we investigated (TCGA and Gravendeel): *HOXD10*, *PDE1C*, *PLS3*, *PTEN *and *TUSC3 *(Table 5). In addition to glioblastoma (grade IV) tumors, Gravendeel *et al. *also characterized 109 grade I to III glioma cases (Table 1). Inclusion of these data in survival analyses made the association with the GNS signature even more apparent (Figure 5b). This is consistent with the above observation that core transcriptional alterations in GNS cells correlate with histological grade of primary tumors. Analysis of data from the studies of Phillips *et al. *[9] and Freije *et al. *[10], which profiled both grade III and IV gliomas (Table 1), further confirmed the correlation between GNS signature and survival (Figure 5b). In summary, the association between GNS signature and patient survival was reproducible in five independent data sets comprising 867 glioma cases in total (Table 1).

**Figure 6:**
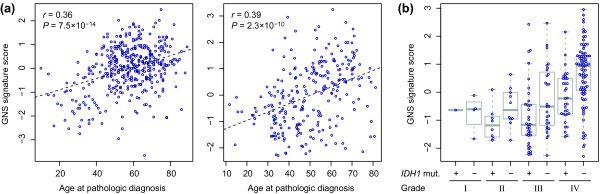
**Association between GNS signature and other survival predictors**. **(a) **Scatter plots demonstrate the correlation between GNS signature score and age at diagnosis for the TCGA (left) and Gravendeel (right) data sets. The regression line, Pearson correlation coefficient (*r*) and *P*-value indicating statistical significance of the correlation are shown. **(b) **GNS signature score for samples in the Gravendeel data set, stratified by *IDH1 *mutation status and histological grade. Blue circles represent individual samples (independent cases) and grey boxplots summarize their distribution. Only cases with known *IDH1 *status are shown (127 mutated, 77 wild type).

We controlled for a range of potential confounding factors; these did not explain the survival trends observed (Additional file 10). Investigating a relationship to known predictors of survival in glioma, we noted that the GNS signature score correlates with patient age at diagnosis, suggesting that the GNS cell-related expression changes are associated with the more severe form of the disease observed in older patients (Figure 6a). Of the genes contributing to the GNS signature, *HOXD10*, *PLS3*, *PTEN *and *TUSC3 *correlated with age both in the TCGA and Gravendeel data sets (Additional file 11).

Most grade III astrocytomas and a minority of glioblastomas carry a mutation affecting codon 132 of the *IDH1 *gene resulting in an amino acid change (R132H, R132S, R132C, R132G, or R132L). The presence of this mutation is associated with lower age at disease onset and better prognosis [47,48]. All 16 GNS cell lines profiled in this study were derived from glioblastoma tumors, and the *IDH1 *locus was sequenced in each cell line (data not shown); none of them harbor the mutation. We therefore investigated whether the GNS signature is characteristic of *IDH1 *wild-type glioblastomas. *IDH1 *status has been determined for most cases in the TCGA and Gravendeel data sets (Table 6) [11,13,17]. As expected, we found that gliomas with the *IDH1 *mutation tend to have lower GNS signature scores than *IDH1 *wild-type gliomas of the same histological grade (Figure 6b). However, we also found the GNS signature to have a stronger survival association than *IDH1 *status (Table 6). The signature remained a significant predictor of patient survival when controlling for *IDH1 *status (Table 6), demonstrating that it contributes independent information to the survival model and does not simply represent a transcriptional state of *IDH1 *wild-type tumors. This was evident in glioblastomas as well as grade I to III gliomas; the effect is thus not limited to grade IV tumors.

**Table 6:** Significance of survival association for GNS signature and *IDH1 *status

		Single covariate		Two covariates	
Data set	Number of cases	GNS signature	*IDH1 *status	GNS signature	*IDH1 *status
TCGA	270	5.3 × 10^-5^	0.0015	0.0091	0.1489
Gravendeel, glioblastoma cases	118	2.7 × 10^-5^	0.0031	9.2 × 10^-4^	0.0840
Gravendeel, grade I to III glioma cases	86	6.5 × 10^-4^	0.5776	6.3 × 10^-4^	0.5408

Wald test *P*-values, indicating association with survival, for each covariate in a Cox proportional hazards model with one or two covariates (GNS signature, *IDH1 *status or both). Cases with unknown *IDH1 *mutation status were excluded.

To investigate whether the correlation between GNS signature and age could be explained by the higher proportion of cases with *IDH1 *mutation among younger patients, we repeated the correlation analysis described above (Figure 6a), limiting the data to glioblastoma cases without *IDH1 *mutation. For the TCGA data set, the correlation was decreased somewhat (Pearson *r *= 0.25 compared to 0.36 for the full data set) but still highly significant (*P *= 6 × 10^-5^), demonstrating that the correlation with age is only partially explained by *IDH1 *status. This result was confirmed in the Gravendeel data set, where the effect of controlling for *IDH1 *status and grade was negligible (*r *= 0.38 compared to 0.39 for the full data set including grade I to III samples). Among the individual signature genes, both *HOXD10 *and *TUSC3 *remained correlated with age in both data sets when limiting the analysis to *IDH1 *wild-type glioblastoma cases (Additional file 11).

### Influence of copy number alterations on the GNS transcriptome

**Figure 7:**
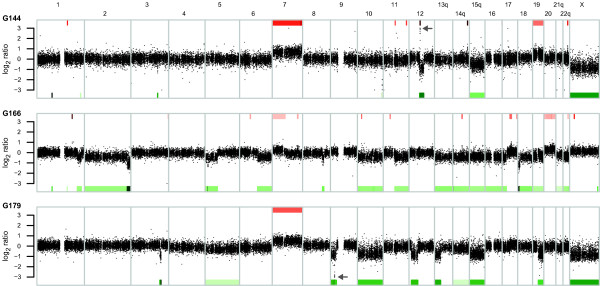
**CGH profiles for GNS lines**. Dots indicate log_2 _ratios for array CGH probes along the genome, comparing each GNS cell line to normal female DNA. Colored segments indicate gain (red) and loss (green) calls, with color intensity proportional to mean log_2 _ratio over the segment. Aberrations known to be common in glioblastoma [11,79] were identified, including gain of chromosome 7 and losses of large parts of chromosomes 10, 13, 14 and 19 in more than one GNS cell line, as well as focal gain of *CDK4 *in G144 (arrow, chromosome 12) and focal loss of the *CDKN2A*-*CDKN2B *locus in G179 (arrow, chromosome 9). The X chromosome was called as lost in G144 and G179 because these two cell lines are from male patients; sex-linked genes were excluded from downstream analyses of aberration calls.

**Figure 8:**
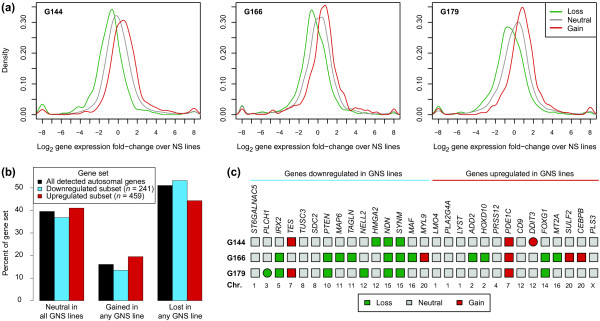
**Copy number changes in GNS cell lines correlate with gene expression levels**. **(a) **Curves show distributions of expression level differences between GNS and NS cells, stratified by aberration calls. The distributions for genes in segments without aberrations (neutral) peak near the 0 mark, corresponding to an equal expression level in GNS and NS cells. Conversely, genes in lost and gained regions tend to be expressed at lower and higher levels, respectively. In each plot, log_2 _fold changes were computed between the indicated GNS cell line and the mean of the two NS cell lines, and capped at (-8, 8) for visualization purposes. To obtain robust fold-change distributions, genes with low expression (<25 tags per million) in both cancer and normal cell types were excluded; consequently, between 6,014 and 6,133 genes underlie each plot. **(b) **For each of the three gene sets listed in the legend (inset), bars represent the percentage of genes with the indicated copy number status. **(c) **Aberration calls for the 29 genes broadly distinguishing GNS from NS cells by qRT-PCR. Circles indicate focal (<10 Mb) aberrations; boxes indicate larger chromosomal segments.

Previous analysis of chromosomal aberrations in G144, G166 and G179 by spectral karyotyping and array CGH detected genetic variants characteristic of glioblastoma [6]. To assess the influence of copy-number changes on the GNS transcriptome, we compared CGH profiles (Figure 7) with Tag-seq data. On a global level, there was an apparent correlation between chromosomal aberrations and gene expression levels (Figure 8a,b), demonstrating that copy-number changes are a significant cause of the observed expression differences. Among the 29 genes differentially expressed between GNS and NS cells in the larger panel assayed by qRT-PCR, there was a tendency for downregulated genes to be lost: 10 out of 15 downregulated genes were in regions of lower than average copy number in one or more GNS cell lines, compared to 4 out of 14 upregulated genes (*P *= 0.046, one-sided Fisher's exact test).

Despite the global correlation between gene expression and copy number, many individual expression changes could not be explained by structural alterations. For example, only a minority of upregulated genes (21%) were located in regions of increased copy number, including whole-chromosome gains (Figure 8b), the survival-associated genes *HOXD10*, *PLS3*, and *TUSC3 *lacked copy-number aberrations consistent with their expression changes, and the survival-associated gene *DDIT3 *was only genetically gained in G144, although highly expressed in all three GNS cell lines (Figure 8c). In general, the 29 genes that robustly distinguish GNS from NS cells did not show a consistent pattern of aberrations: only three genes (*PDE1C*, *NDN *and *SYNM*) were located in regions similarly affected by genetic lesions in all lines. Thus, in addition to copy-number alterations, other factors are important in shaping the GNS transcriptome, and regulatory mechanisms may differ among GNS cell lines yet produce similar changes in gene expression.

## Discussion

To reveal transcriptional changes that underlie glioblastoma, we performed an in-depth analysis of gene expression in malignant stem cells derived from patient tumors in relation to untransformed, karyotypically normal NS cells. These cell types are closely related and it has been hypothesized that gliomas arise by mutations in NS cells or in glial cells that have reacquired stem cell features [2]. We measured gene expression by high-throughput RNA tag sequencing (Tag-seq), a method that features high sensitivity and reproducibility compared to microarrays [7]. qRT-PCR validation further demonstrates that Tag-seq expression values are highly accurate. Other cancer samples and cell lines have recently been profiled with the same method [8,47] and it should be feasible to directly compare those results to the data presented here.

Through Tag-seq expression profiling of normal and cancer stem cells followed by qRT-PCR validation in a wider panel of 22 cell lines, we identified 29 genes strongly discriminating GNS from NS cells. Some of these genes have previously been implicated in glioma, including four with a role in adhesion and/or migration, *CD9*, *ST6GALNAC5*, *SYNM *and *TES *[49-52], and two transcriptional regulators, *FOXG1 *and *CEBPB. FOXG1*, which has been proposed to act as an oncogene in glioblastoma by suppressing growth-inhibitory effects of transforming growth factor β [53], showed remarkably strong expression in all 16 GNS cell lines assayed by qRT-PCR. *CEBPB *was recently identified as a master regulator of a mesenchymal gene expression signature associated with poor prognosis in glioblastoma [45]. Studies in hepatoma and pheochromocytoma cell lines have shown that the transcription factor encoded by *CEBPB *(C/EBPβ) promotes expression of *DDIT3 *[54], another transcriptional regulator that we found to be upregulated in GNS cells. *DDIT3 *encodes the protein CHOP, which in turn can inhibit C/EBPβ by dimerizing with it and acting as a dominant negative [54]. This interplay between *CEBPB *and *DDIT3 *may be relevant for glioma therapy development, as *DDIT3 *induction in response to a range of compounds sensitizes glioma cells to apoptosis (see, for example, [55]).

Our results also corroborate a role in glioma for several other genes with limited prior links to the disease. This list includes *PLA2G4A*, *HMGA2*, *TAGLN *and *TUSC3*, all of which have been implicated in other neoplasias (Additional file 12). *PLA2G4A *encodes a phospholipase that functions in the production of lipid signaling molecules with mitogenic and pro-inflammatory effects. In a subcutaneous xenograft model of glioblastoma, expression of *PLA2G4A *by the host mice was required for tumor growth [56]. For *HMGA2*, a transcriptional regulator downregulated in most GNS cell lines, low or absent protein expression has been observed in glioblastoma compared to low-grade gliomas [57], and *HMGA2 *polymorphisms have been associated with survival time in glioblastoma [58]. The set of 29 genes found to generally distinguish GNS from NS cells also includes multiple genes implicated in other neoplasias, but without direct links to glioma (Additional file 12). Of these, the transcriptional regulator *LMO4*, may be of particular interest, as it is well studied as an oncogene in breast cancer and regulated through the phosphoinositide 3-kinase pathway [59], which is commonly affected in glioblastoma [11].

Five of these 29 genes have not been directly implicated in cancer. This list comprises one gene downregulated in GNS cells (*PLCH1*) and four upregulated (*ADD2*, *LYST*, *PDE1C *and *PRSS12*). *PLCH1 *is involved in phosphoinositol signaling [60], like the frequently mutated phosphoinositide 3-kinase complex [11]. *ADD2 *encodes a cytoskeletal protein that interacts with FYN, a tyrosine kinase promoting cancer cell migration [61,62]. For *PDE1C*, a cyclic nucleotide phosphodiesterase gene, we found higher expression to correlate with shorter survival after surgery. Upregulation of *PDE1C *has been associated with proliferation in other cell types through hydrolysis of cAMP and cGMP [63,64]. *PRSS12 *encodes a protease that can activate tissue plasminogen activator (tPA) [65], an enzyme that is highly expressed by glioma cells and has been suggested to promote invasion [66].

By considering expression changes in a pathway context, we identified additional candidate glioblastoma genes, such as the putative cell adhesion gene *ITGBL1 *[67], the orphan nuclear receptor *NR0B1*, which is strongly upregulated in G179 and is known to be upregulated and mediate tumor growth in Ewing's sarcoma [68], and the genes *PARP3 *and *PARP12*, which belong to the poly(ADP-ribose) polymerase (PARP) family of ADP-ribosyl transferase genes involved in DNA repair (Table 4). The upregulation of these PARP genes in GNS cells may have therapeutic relevance, as inhibitors of their homolog *PARP1 *are in clinical trials for brain tumors [69].

Transcriptome analysis thus identified multiple genes of known significance in glioma pathology as well as several novel candidate genes and pathways. These results are further corroborated by survival analysis, which revealed a GNS expression signature associated with patient survival time in five independent data sets. This finding is compatible with the notion that gliomas contain a GNS component of relevance for prognosis. Five individual GNS signature genes were significantly associated with survival of glioblastoma patients in both of the two largest data sets: *PLS3*, *HOXD10*, *TUSC3*, *PDE1C *and the well-studied tumor suppressor *PTEN. PLS3 *(T-plastin) regulates actin organization and its overexpression in the CV-1 cell line resulted in partial loss of adherence [70]. Elevated *PLS3 *expression in GNS cells may thus be relevant for the invasive phenotype. The association between transcriptional upregulation of *HOXD10 *and poor survival is surprising, because HOXD10 protein levels are suppressed by a microRNA (miR-10b) highly expressed in gliomas, and it has been suggested that HOXD10 suppression by miR-10b promotes invasion [71]. Notably, the *HOXD10 *mRNA upregulation we observe in GNS cells also occurs in glioblastoma tumors, as shown by comparison with grade III astrocytoma (Figure 3b). Similarly, miR-10b is present at higher levels in glioblastoma compared to gliomas of lower grade [71]. It is conceivable that *HOXD10 *transcriptional upregulation and post-transcriptional suppression is indicative of a regulatory program associated with poor prognosis in glioma.

Tumors from older patients featured an expression pattern more similar to the GNS signature. One of the genes contributing to this trend, *TUSC3*, is known to be silenced by promoter methylation in glioblastoma, particularly in patients aged over 40 years [72]. Loss or downregulation of *TUSC3 *has been found in other cancers, such as of the colon, where its promoter becomes increasingly methylated with age in the healthy mucosa [73]. Taken together, these data suggest that transcriptional changes in healthy aging tissue, such as *TUSC3 *silencing, may contribute to the more severe form of glioma in older patients. Thus, the molecular mechanisms underlying the expression changes described here are likely to be complex and varied. To capture these effects and elucidate their causes, transcriptome analysis of cancer samples will benefit from integration of diverse genomic data, including structural and nucleotide-level genetic alterations, as well as DNA methylation and other chromatin modifications.

To identify expression alterations common to most glioblastoma cases, other studies have profiled tumor resections in relation to non-neoplastic brain tissue [47,74,75]. While such comparisons have been revealing, their power is constrained by discrepancies between reference and tumor samples - for instance, the higher neuronal content of normal brain tissue compared to tumors. Gene expression profiling of tumor tissue further suffers from mixed signal due to a stromal cell component and heterogeneous populations of cancer cells, only some of which contribute to tumor progression and maintenance [2]. Part of a recent study bearing a closer relationship to our analysis examined gene expression in another panel of glioma-derived and normal NS cells [76], but included neurosphere cultures, which often contain a heterogeneous mixture of self-renewing and differentiating cells.

Here, we have circumvented these issues by profiling uniform cultures of primary malignant stem cell lines that can reconstitute the tumor *in vivo *[6], in direct comparison to normal counterparts of the same fundamental cell type [4,5]. While the resulting expression patterns largely agree with those obtained from glioblastoma tissues, there are notable differences. For example, we found the breast cancer oncogene *LMO4 *(discussed above) to be upregulated in most GNS cell lines, although its average expression in glioblastoma tumors is low relative to normal brain tissue (Figure 3a). Similarly, *TAGLN *and *TES *were absent or low in most GNS cell lines, but displayed the opposite trend in glioblastoma tissue compared to normal brain (Figure 3c) or grade III astrocytoma (Figure 3d). Importantly, both *TAGLN *and *TES *have been characterized as tumor suppressors in malignancies outside the brain and the latter is often silenced by promoter hypermethylation in glioblastoma [77,78].

## Conclusions

Our results support the use of GNS cells as a relevant model for investigating the molecular basis of glioblastoma, and the use of NS cell lines as controls in this setting. Transcriptome sequencing revealed aberrant gene expression patterns in GNS cells and defined a molecular signature of the proliferating cell population that drives malignant brain cancers. These transcriptional alterations correlate with several prognostic indicators and are strongly associated with patient survival in both glioblastoma and lower-grade gliomas, suggesting that a greater GNS cell component contributes to poorer prognosis. Several genes observed to be consistently altered in GNS cells have not previously been implicated in glioma, but are known to play a role in other neoplasias or in cellular processes related to malignancy. Such alterations include changes in oncogene and tumor suppressor expression not detectable by microarray profiling of post-surgical glioma biopsies. These findings demonstrate the utility of cancer stem cell models for advancing the molecular understanding of tumorigenesis.

## Abbreviations

CGH, comparative genomic hybridization; DMEM, Dulbecco's modified Eagle's medium; FDR, false discovery rate; GNS, glioma neural stem; MHC, major histocompatibility complex; NS, neural stem; nt, nucleotide; PARP, poly(ADP-ribose) polymerase; qRT-PCR, quantitative reverse-transcription polymerase chain reaction; RNA-seq, high-throughput shotgun sequencing of RNA transcripts; SAGE, serial analysis of gene expression; Tag-seq, high-throughput sequencing of transcript tags; TCGA, The Cancer Genome Atlas.

## Competing interests

The authors declare that they have no competing interests.

## Authors' contributions

PGE analyzed data, designed experiments and drafted the manuscript. DT analyzed data and helped draft the manuscript. SHS and CE performed experiments. PB and SMP conceived and supervised the study and participated in writing the manuscript. All authors read and approved the final content.

## Supplementary Material

Additional file 1 **Supplemental methods**. Detailed method descriptions for (1) assignment of tags to genes, (2) differential expression analysis of Tag-seq data, and (3) construction of the integrated glioma pathway map. Format: PDF. Click here for file

Additional file 2 **Classification of sequenced tags**. Table listing the number of sequenced tags in each sample and the proportion of tags assigned to different categories by the Tag-seq data processing pipeline. Format: XLS. Click here for file

Additional file 3 **qRT-PCR data**. Table with raw and normalized C*_t _*values from TaqMan qRT-PCR assays. Format: XLS. Click here for file

Additional file 4 **TCGA sample IDs**. Table listing sample identifiers for the TCGA expression and survival data used. Format: XLS. Click here for file

Additional file 5 **Differentially expressed genes at 10% FDR**. Table with expression values, fold-changes and *P*-values for the genes found to be expressed at a higher or lower average level in GNS cells compared to NS cells by Tag-seq (10% FDR). Format: XLS. Click here for file

Additional file 6 **Differentially expressed non-coding RNAs**. Table of non-coding RNAs found to be differentially expressed between GNS and NS cells by Tag-seq. Format: XLS. Click here for file

Additional file 7 **Integrated pathway map**. Network diagram of the integrated glioma pathway, with differentially expressed genes colored according to fold-change between GNS and NS cells. Format: PDF. Click here for file

Additional file 8 **Core differentially expressed genes**. Table of genes with large expression changes common to the GNS cell lines G144, G166 and G179, relative to the normal NS cell lines CB541 and CB660. Format: XLS. Click here for file

Additional file 9 **Kaplan-Meier plots for multiple GNS signature score thresholds**. Survival curves illustrating the association between GNS signature and patient survival for three independent glioblastoma data sets and a range of percentile thresholds on GNS signature score. Format: PDF. Click here for file

Additional file 10 **Controls for survival tests on GNS expression signature**. Text and table detailing how confounding factors were controlled for when testing for an association between the GNS expression signature and patient survival. Format: XLS. Click here for file

Additional file 11 **Correlation between age at diagnosis and GNS signature gene expression**. Scatter plots demonstrating the correlation between age at diagnosis and expression of GNS signature genes. Format: PDF. Click here for file

Additional file 12 **Disease association of GNS cell-specific genes**. Literature survey for the set of 29 genes found to distinguish GNS from NS cells across a panel of 22 different lines. The table details whether each gene has previously been implicated in glioma or other neoplasias, and includes references to relevant publications. Format: XLS. Click here for file
