# Supplementary material for: Digital transcriptome profiling of normal and glioblastoma-derived neural stem cells identifies genes associated with patient survival
Source: Genome Med. 2012 Oct 9;4(10):76. doi: 10.1186/gm377 (PMC3556652; doi:10.1186/gm377)
Supplement: Additional file 1 — Supplemental methods. Detailed method descriptions for (1) assignment of tags to genes, (2) differential expression analysis of Tag-seq data, and (3) construction of the integrated glioma pathway map. Format: PDF. [file gm377-S1.PDF]

Supplemental Methods for

Digital transcriptome profiling of normal and glioblastoma-derived neural stem cells identifies genes associated with patient survival

Pär G. Engström, Diva Tommei, Stefan H. Stricker, Christine Ender, Steven M. Pollard and Paul Bertone

Assignment of tags to genes

To assign tags to genes, we employed a hierarchical strategy based on the expectation that tags are most likely to originate from the 3′-most NlaIII site in known transcripts. To this end, virtual tags were extracted from SAGE Genie [1] and Ensembl [2]. The SAGE Genie annotation (version Feb. 2, 2010) consisted of 105 sets of virtual tags obtained by scanning for NlaIII sites in cDNAs (from RefSeq, MGC and GenBank), expressed sequence tags (ESTs), UniGene consensus sequences and transcribed regions identified by tiling array analysis (transfrags). SAGE Genie further classifies these virtual tag sets based on the position of the tag relative to the transcript 3′-end, along with indicators of 3′-end reliability (polyadenylation signal and poly(A) tail).

Since SAGE Genie does not cover Ensembl transcripts, we also extracted virtual tags from Ensembl genes of biotype 'protein_coding' or 'processed_transcript'. A virtual tag was extracted from the 3′-most NlaIII site in each Ensembl transcript. If this site was located fewer than 17 nt from the end of the transcript, the sequence was extended with As to represent the poly(A) tail. In such cases, additional upstream tags were also extracted until one tag fully contained in the transcript sequence was obtained.

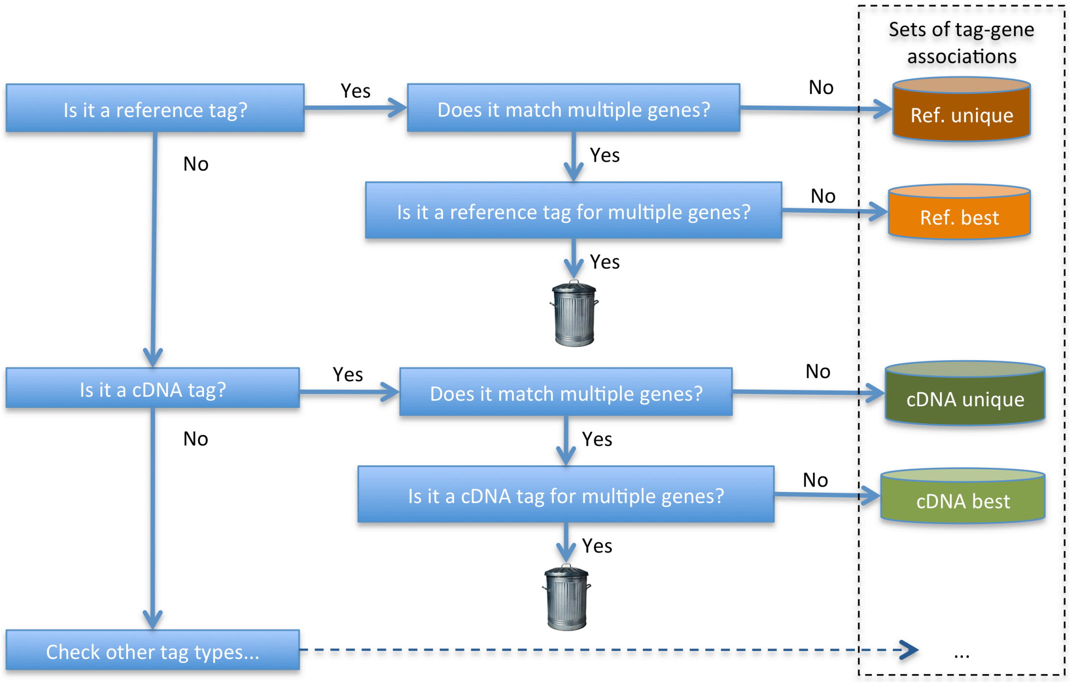

Hierarchical strategy for assigning tags to genes.

Some virtual tags can be associated with multiple loci, in particular when considering the more comprehensive SAGE Genie annotations based on ESTs. To prioritize more likely tag-gene associations, we assigned virtual tags to genes using the hierarchical strategy schematically illustrated on page 1. Virtual tags were initially ranked as follows:

1. Reference tags: SAGE Genie tags from the 3′-most cut site in RefSeq or MGC cDNAs having a poly(A) tail or a polyadenylation signal, and Ensembl tags from transcripts of type 'protein_coding' or 'non_coding'.

2. cDNA tags: SAGE Genie tags from the 3′-most cut site in GenBank cDNAs having a poly(A) tail or a polyadenylation signal, and remaining Ensembl tags.

Remaining SAGE Genie datasets were assigned ranks from 3 onwards, following the ranking order in SAGE Genie. We used all SAGE Genie mappings except those to transfrags. Each virtual tag was also assigned an ambiguity status: "unique" if the tag mapped to a single gene; or "best" if the tag mapped to multiple genes, but its highest-ranking mappings represented a single gene. We discarded virtual tags that had multiple highest-ranking mappings unless they all corresponded to the same gene. To determine whether different tag mappings identified the same gene, we made use of cross-references between Ensembl Gene IDs, Entrez Gene IDs, UniGene IDs and cDNA/EST accession numbers, as well as genomic coordinates (obtained from the Ensembl and UCSC Genome Browser databases) of Ensembl Genes, RefSeq transcripts, cDNAs and ESTs.

Multiple virtual tag sets suitable for different analyses were obtained by thresholding on rank. In addition, the short-read aligner *bowtie* [3] was applied to determine unique, perfect matches for sequenced tags to the reference genome.

Tag-seq differential expression analysis

The Tag-seq data were used to compare gene expression between the three GNS cell lines G144, G166 and G179 and the two NS cell lines CB541 and CB660, leading to the identification of three sets of differentially expressed genes:

- 739 genes (Additional file 5) with different mean expression between GNS and NS cells were identified at an estimated false discovery rate (FDR) of 10%, using the reference virtual tags (rank 1) with ambiguity status "unique" or "best". This is a conservative set of virtual tags, but covers the great majority of sequence reads that could be mapped to the genome or known transcriptome (Additional file 2), and should therefore be suitable for most analyses. We calculated an expression value for each gene in each cell line by summing the counts of tags assigned to the gene. The Bioconductor package *DESeq* was then used to call differentially expressed genes, as detailed below. The results were used for pathway analysis, functional enrichment analysis and comparison with copy number aberrations.
- 92 genes (Additional file 8) displaying major expression differences common to all three GNS cell lines were identified using all tags mapped to the transcriptome or genome. Differential expression analysis was carried out using four different tag sets independently and the results combined. The tag

sets were: (a) rank 1 virtual tags; (b) rank 1 and 2 virtual tags; (c) all virtual tags; and (d) all observed tags mapping perfectly to a unique location in the reference genome. For tag sets a–c, gene expression values were first computed by summing the counts of tags assigned to each gene. For set d, individual tags were tested for differential expression. In each case, statistical testing was carried out using *DESeq* at an FDR threshold of 1% and additionally requiring: a two-fold or greater change in each GNS line compared to each NS line, with the direction of change being consistent among the lines; and an expression level above 30 tags per million in each GNS line (if upregulated in GNS lines) or each NS line (if downregulated in GNS lines).

- 25 noncoding RNAs with different mean expression between GNS and NS lines were identified at an estimated FDR of 10%, by testing each tag that mapped perfectly to a unique location in the reference genome (using *DESeq* as described below) and filtering the results to exclude protein-coding transcripts. All 25 RNAs are supported by published cDNA sequences or expressed sequence tags (Additional file 6).

The Bioconductor package *DESeq* version 1.0.6 was used to normalize expression values and test for differences in expression between GNS and NS cells, by applying the functions `estimateSizeFactors`, `estimateVarianceFunctions` and `nbinomTest` with default parameters. This procedure implements the methodology described by Anders and Huber [4]. Briefly, the normalization serves to make tag counts comparable between samples by adjusting for differences in read depth and transcriptome complexity. The variance within each group of samples (GNS and NS) is then estimated by fitting a negative binomial model to the data, and this model is used to test each gene (or tag) for difference in expression between the two groups.

Integrated glioma pathway map

Pathway maps were created in Cytoscape 2.8.2 [5], integrating data from the following sources:

- The glioma and P53 pathways from the KEGG database [6]
- The glioma pathways described by Cerami et al. [7], Furnari et al. [8] and GenWay Biotech [9]
- The P53 pathway from the Panther database [10] and the GeneAssist Pathway Atlas [11]
- The P53, PTEN and Rb1 pathways from Biocarta [12]
- The MAPK signaling pathways from the Cell Signaling Technology website [13] and Wikipedia [14]

Every interaction in the integrated pathway was checked to be validated experimentally via literature searches and the interaction databases BioGRID [15] and IntAct [16].

References

1. Boon K, Osorio EC, Greenhut SF, Schaefer CF, Shoemaker J, Polyak K, Morin PJ, Buetow KH, Strausberg RL, De Souza SJ, Riggins GJ: **An anatomy of normal**

and malignant gene expression. *Proc Natl Acad Sci USA* 2002, **99**:11287–11292.

2. Flicek P, Amode MR, Barrell D, Beal K, Brent S, Carvalho-Silva D, Clapham P, Coates G, Fairley S, Fitzgerald S, Gil L, Gordon L, Hendrix M, Hourlier T, Johnson N, Kähäri AK, Keefe D, Keenan S, Kinsella R, Komorowska M, Koscielny G, Kulesha E, Larsson P, Longden I, McLaren W, Muffato M, Overduin B, Pignatelli M, Pritchard B, Riat HS, *et al.*: **Ensembl 2012**. *Nucleic Acids Res* 2012, **40**:D84–90.
3. Langmead B, Trapnell C, Pop M, Salzberg S: **Ultrafast and memory-efficient alignment of short DNA sequences to the human genome**. *Genome Biol* 2009, **10**:R25.
4. Anders S, Huber W: **Differential expression analysis for sequence count data**. *Genome Biol* 2010, **11**:R106.
5. Smoot ME, Ono K, Ruscheinski J, Wang P-L, Ideker T: **Cytoscape 2.8: new features for data integration and network visualization**. *Bioinformatics* 2011, **27**:431–432.
6. Kanehisa M, Goto S, Sato Y, Furumichi M, Tanabe M: **KEGG for integration and interpretation of large-scale molecular data sets**. *Nucleic Acids Res* 2011.
7. Cerami E, Demir E, Schultz N, Taylor BS, Sander C: **Automated network analysis identifies core pathways in glioblastoma**. *PLoS ONE* 2010, **5**:e8918.
8. Furnari FB, Fenton T, Bachoo RM, Mukasa A, Stommel JM, Stegh A, Hahn WC, Ligon KL, Louis DN, Brennan C, Chin L, DePinho RA, Cavenee WK: **Malignant astrocytic glioma: genetics, biology, and paths to treatment**. *Genes Dev* 2007, **21**:2683–2710.
9. **GenWay Biotech: Glioma pathway** [http://legacy.genwaybio.com/gw_file.php?fid=2519].
10. Mi H, Dong Q, Muruganujan A, Gaudet P, Lewis S, Thomas PD: **PANTHER version 7: improved phylogenetic trees, orthologs and collaboration with the Gene Ontology Consortium**. *Nucleic Acids Res* 2010, **38**:D204–210.
11. **GeneAssist Pathway Atlas** [http://www5.appliedbiosystems.com/tools/pathway/all_pathway_list.php].
12. **Biocarta** [http://www.biocarta.com].
13. **Cell Signaling Technology: MAPK/Erk in Growth and Differentiation** [http://www.cellsignal.com/reference/pathway/MAPK_ERK_Growth.html].
14. **Wikipedia: MAPK pathway diagram** [http://en.wikipedia.org/wiki/File:MAPKpathway.jpg].
15. Stark C, Breitkreutz B-J, Chatr-Aryamontri A, Boucher L, Oughtred R, Livstone MS, Nixon J, Van Auken K, Wang X, Shi X, Reguly T, Rust JM, Winter A, Dolinski K, Tyers M: **The BioGRID Interaction Database: 2011 update**. *Nucleic Acids Res* 2011, **39**:D698–704.
16. Aranda B, Achuthan P, Alam-Faruque Y, Armean I, Bridge A, Derow C, Feuermann M, Ghanbarian AT, Kerrien S, Khadake J, Kerssemakers J, Leroy C, Menden M, Michaut M, Montecchi-Palazzi L, Neuhauser SN, Orchard S, Perreau V, Roechert B, van Eijk K, Hermjakob H: **The IntAct molecular interaction database in 2010**. *Nucleic Acids Res* 2010, **38**:D525–531.
