# Supplementary material for: Digital transcriptome profiling of normal and glioblastoma-derived neural stem cells identifies genes associated with patient survival
Source: Genome Med. 2012 Oct 9;4(10):76. doi: 10.1186/gm377 (PMC3556652; doi:10.1186/gm377)
Supplement: Additional file 7 — Integrated pathway map. Network diagram of the integrated glioma pathway, with differentially expressed genes colored according to fold-change between GNS and NS cells. Format: PDF. [file gm377-S7.PDF]

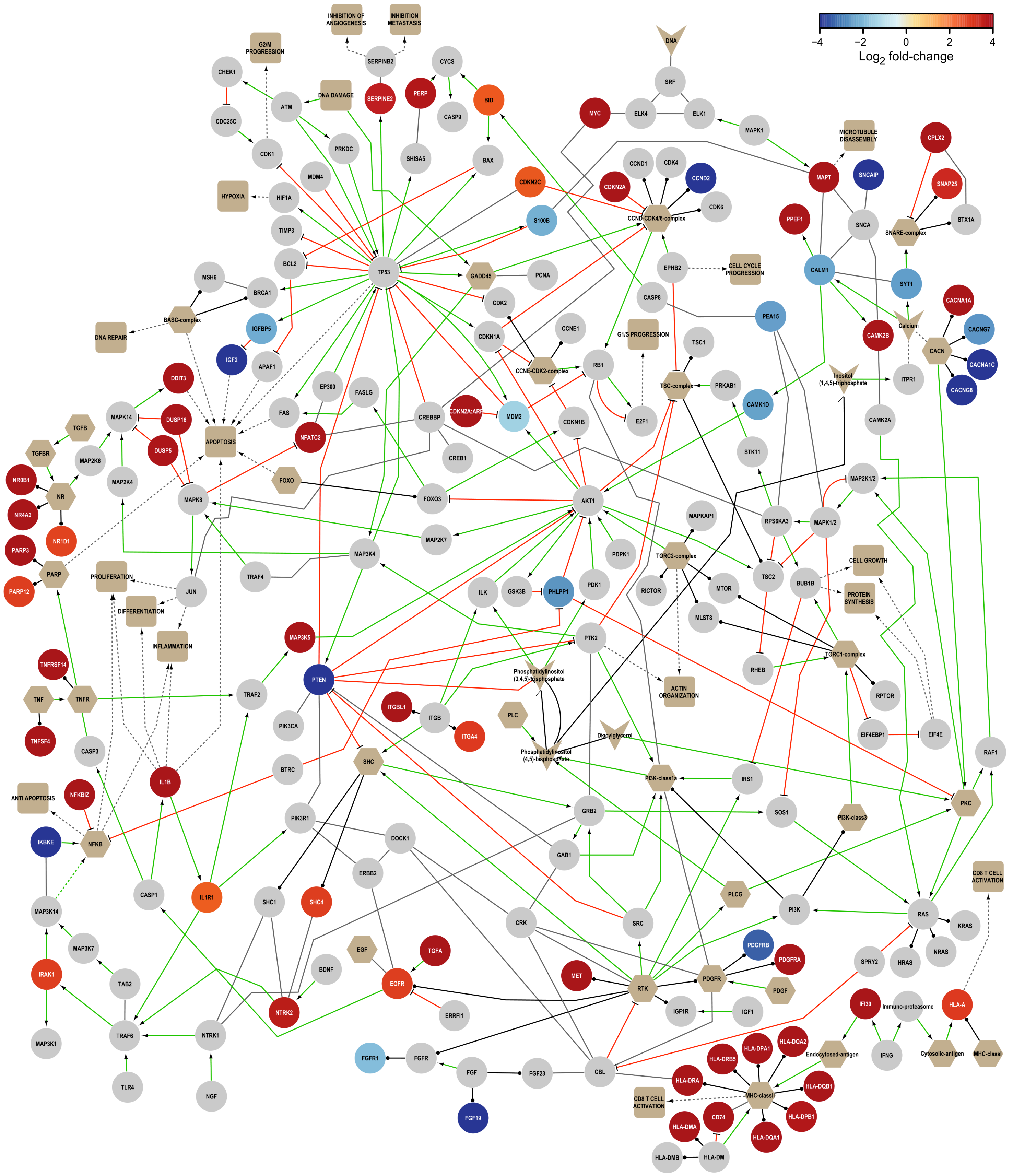

Integrated pathway map. *De novo* assembled pathway composed of 239 nodes, including known glioma-affected pathways as well as relevant proliferation, cell survival and apoptosis pathways. There are 182 nodes representing individual genes, depicted as circles and colored according to expression fold-change between the GNS and NS lines (see color key), or grey unless the expression difference was statistically significant at 10% FDR. Gene complexes and families (hexagons), small molecules (chevrons) and affected cellular processes (squares) are also included. Edges indicate *activation* (green), *inhibition* (red), *contains* (black with circular tip), *becomes* (black with half arrow) and other interactions (grey).
