# Supplementary material for: Digital transcriptome profiling of normal and glioblastoma-derived neural stem cells identifies genes associated with patient survival
Source: Genome Med. 2012 Oct 9;4(10):76. doi: 10.1186/gm377 (PMC3556652; doi:10.1186/gm377)
Supplement: Additional file 9 — Kaplan-Meier plots for multiple GNS signature score thresholds. Survival curves illustrating the association between GNS signature and patient survival for three independent glioblastoma data sets and a range of percentile thresholds on GNS signature score. Format: PDF. [file gm377-S9.PDF]

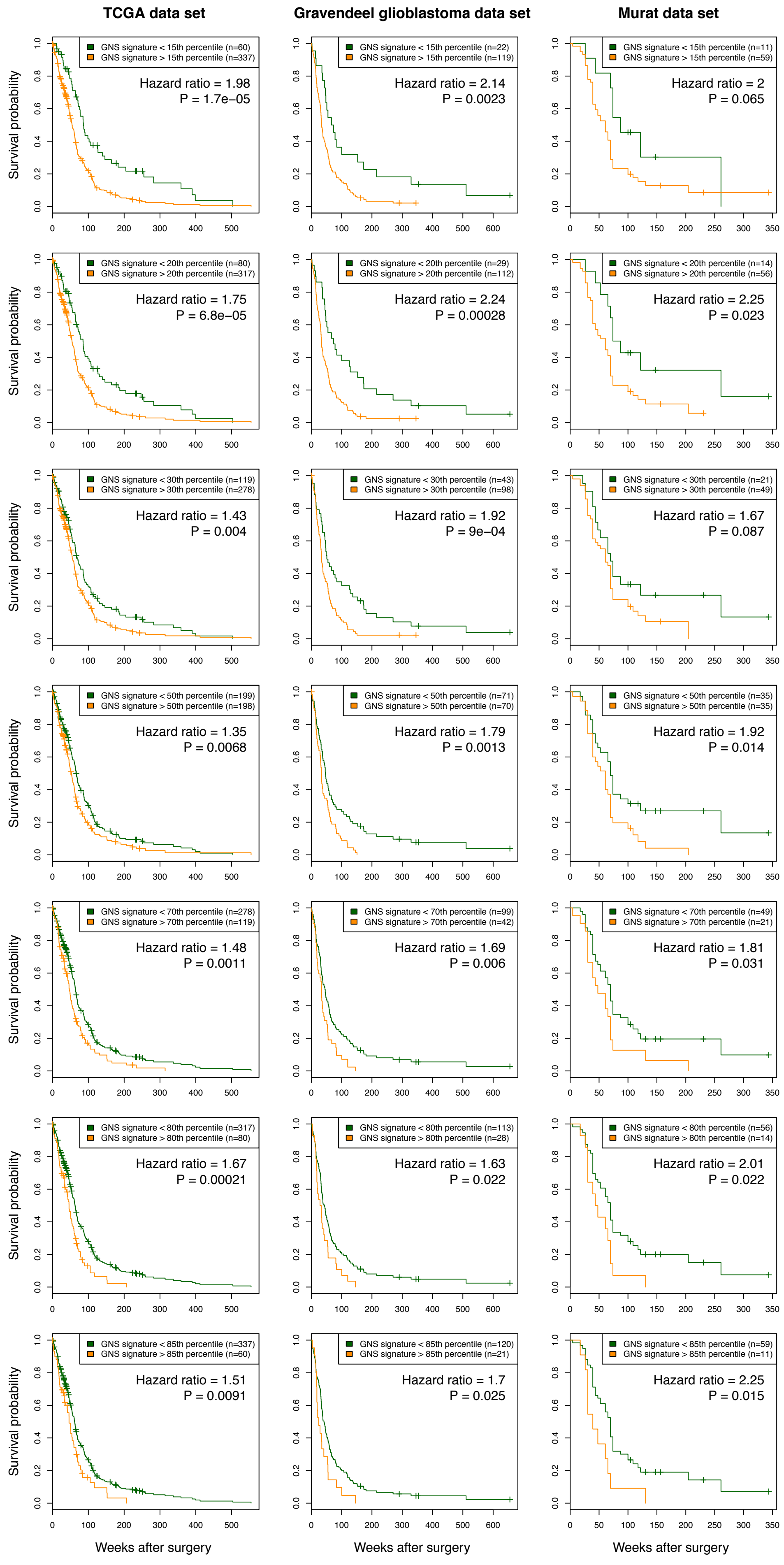

Kaplan-Meier plots for multiple GNS signature score thresholds. Survival curves illustrate the association between GNS signature and patient survival for three independent glioblastoma data sets and a range of percentile thresholds on GNS signature score (from top: 15th, 20th, 30th, 50th, 70th, 80th and 85th percentile). Higher scores indicate greater similarity to the GNS expression profile. Hazard ratios and log-rank *P*-values were computed by fitting a Cox proportional hazards model to the data.
