# Supplementary material for: Digital transcriptome profiling of normal and glioblastoma-derived neural stem cells identifies genes associated with patient survival
Source: Genome Med. 2012 Oct 9;4(10):76. doi: 10.1186/gm377 (PMC3556652; doi:10.1186/gm377)
Supplement: Additional file 11 — Correlation between age at diagnosis and GNS signature gene expression. Scatter plots demonstrating the correlation between age at diagnosis and expression of GNS signature genes. Format: PDF. [file gm377-S11.PDF]

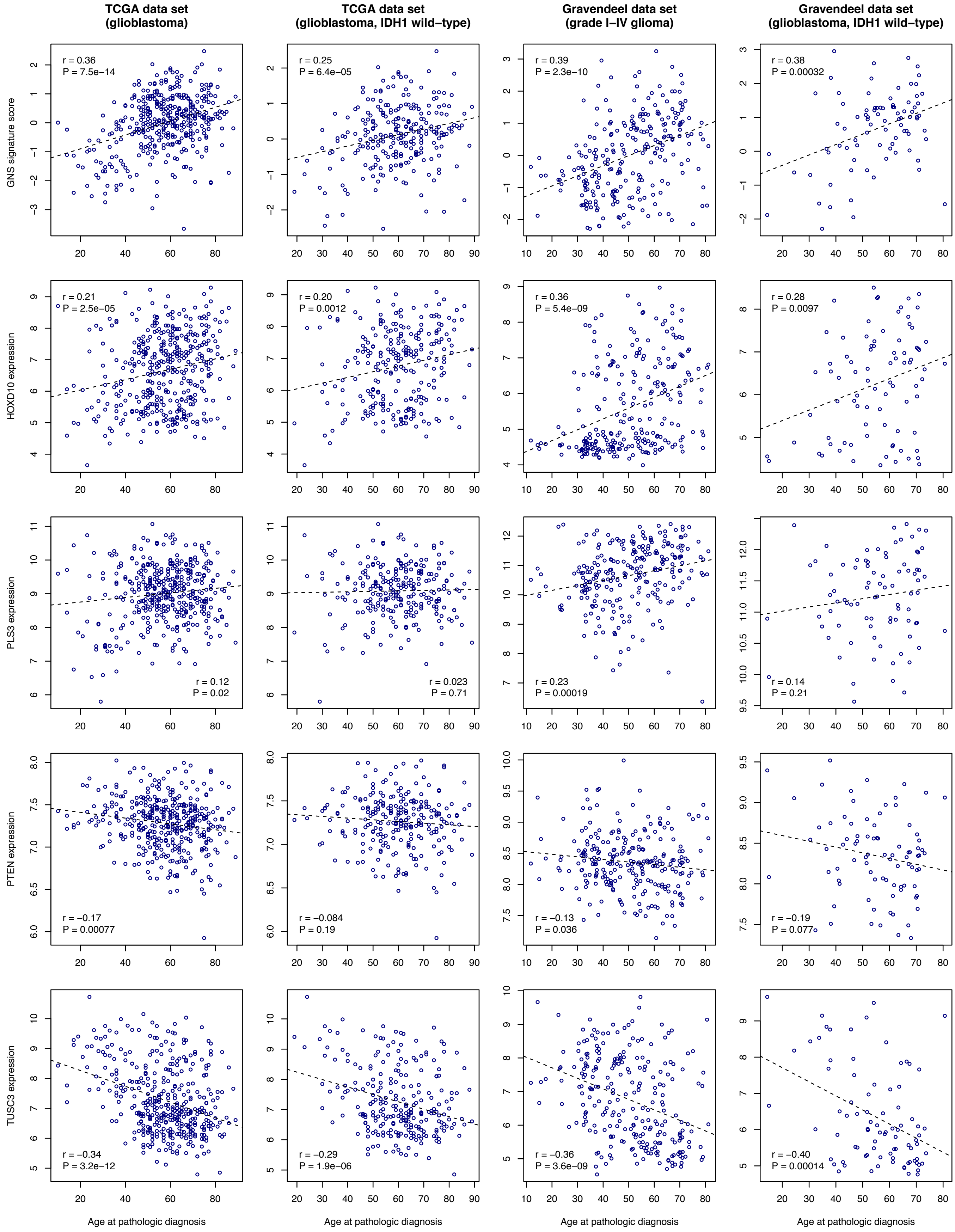

Correlation between age at diagnosis and expression of GNS signature genes. Age versus gene expression is plotted for the GNS signature and four signature genes that displayed a significant correlation with age ($P < 0.05$) in both the TCGA and Gravendeel data sets. The full TCGA and Gravendeel data sets are shown, as well as subsets limited to glioblastoma tumors without *IDH1* mutation. The regression line, Pearson *r* (correlation coefficient) and *P*-value indicating statistical significance of the correlation are shown.
